# Variational modularity at the cell level: insights from the sperm head of the house mouse

**DOI:** 10.1186/1471-2148-13-179

**Published:** 2013-09-03

**Authors:** Nuria Medarde, Francesc Muñoz-Muñoz, María José López-Fuster, Jacint Ventura

**Affiliations:** 1 Departament de Biologia Animal, Biologia Vegetal i Ecologia, Facultat de Biociències, Universitat Autònoma de Barcelona, Cerdanyola del Vallès 08193, Spain; 2 Departament de Biologia Animal, IRBio, Institut de Recerca de Biodiversitat, Facultat de Biologia, Universitat de Barcelona, Avgda. Diagonal, 643, Barcelona 08028, Spain

**Keywords:** Variational modularity, Sperm morphology, Geometric morphometrics, *Mus musculus domesticus*, Robertsonian system

## Abstract

**Background:**

Modularity is an important feature in the evolvability of organisms, since it allows the occurrence of complex adaptations at every single level of biological systems. While at the cellular level the modular organization of molecular interactions has been analyzed in detail, the phenotypic modularity (or variational modularity) of cell shape remains unexplored. The mammalian spermatozoon constitutes one of the most complex and specialized cell types found in organisms. The structural heterogeneity found in the sperm head suggests an association between its inner composition, shape and specificity of function. However, little is known about the extent of the connections between these features. Taking advantage of the house mouse sperm morphology, we analyzed the variational modularity of the sperm head by testing several hypotheses related to its structural and functional organization. Because chromosomal rearrangements can affect the genotype-phenotype map of individuals and thus modify the patterns of covariation between traits, we also evaluate the effect of Robertsonian translocations on the modularity pattern of the sperm head.

**Results:**

The results indicated that the house mouse sperm head is divided into three variational modules (the acrosomal, post-acrosomal and ventral spur module), which correspond to the main regions of the cytoskeletal mesh beneath the plasma membrane, i.e., the perinuclear theca. Most of the covariation is concentrated between the ventral spur and the acrosomal and post-acrosomal modules. Although the Rb fusions did not alter the main modularity pattern, they did affect the percentages of covariation between pairs of modules.

**Conclusions:**

The structural heterogeneity of the cytoskeleton is responsible for the modular organization of the sperm head shape, corroborating the role that this structure has in maintaining the cell shape. The reduction in percentages of shape covariation between pairs of modules in Rb sperms suggests that chromosomal rearrangements could induce changes in the genotype-phenotype map. Nevertheless, how these variations affect sperm fertilization success is yet to be elucidated.

## Background

Organisms are composed of elements that, although coordinated, show obvious signs of heterogeneity with respect to certain kinds of processes [1,2]. These elements, called modules, are internally integrated but relatively independent of one another [2]. Thus, modularity is considered a key feature of biological organization that allows the modification of certain parts of organisms with minor effects on other parts, thereby contributing to evolvability [3]. Modularity occurs at every single level of biological organization, from molecular interactions to networks of ecological connections [1,2]. Variational modularity (that is, groups of correlated characters) has long been recognized in morphological traits [1,2] since it provides the evolutionary flexibility required to induce adaptive changes in certain regions of complex phenotypic structures. At the cell level, the structural and functional modularity of molecular networks have been studied in detail [4,5], but to our knowledge, the variational modularity of cell morphology has not been examined to date. The relations between different kinds of modularity in biological organization are still not well understood, and their comparative study may provide insights into evolutionary processes [6]. The male gametes of certain mammals may represent an ideal model for testing the connections between different kinds of modularity, as they are highly polarized cells with structurally and functionally differentiated regions that are morphologically recognizable [7].

**Figure 1:**
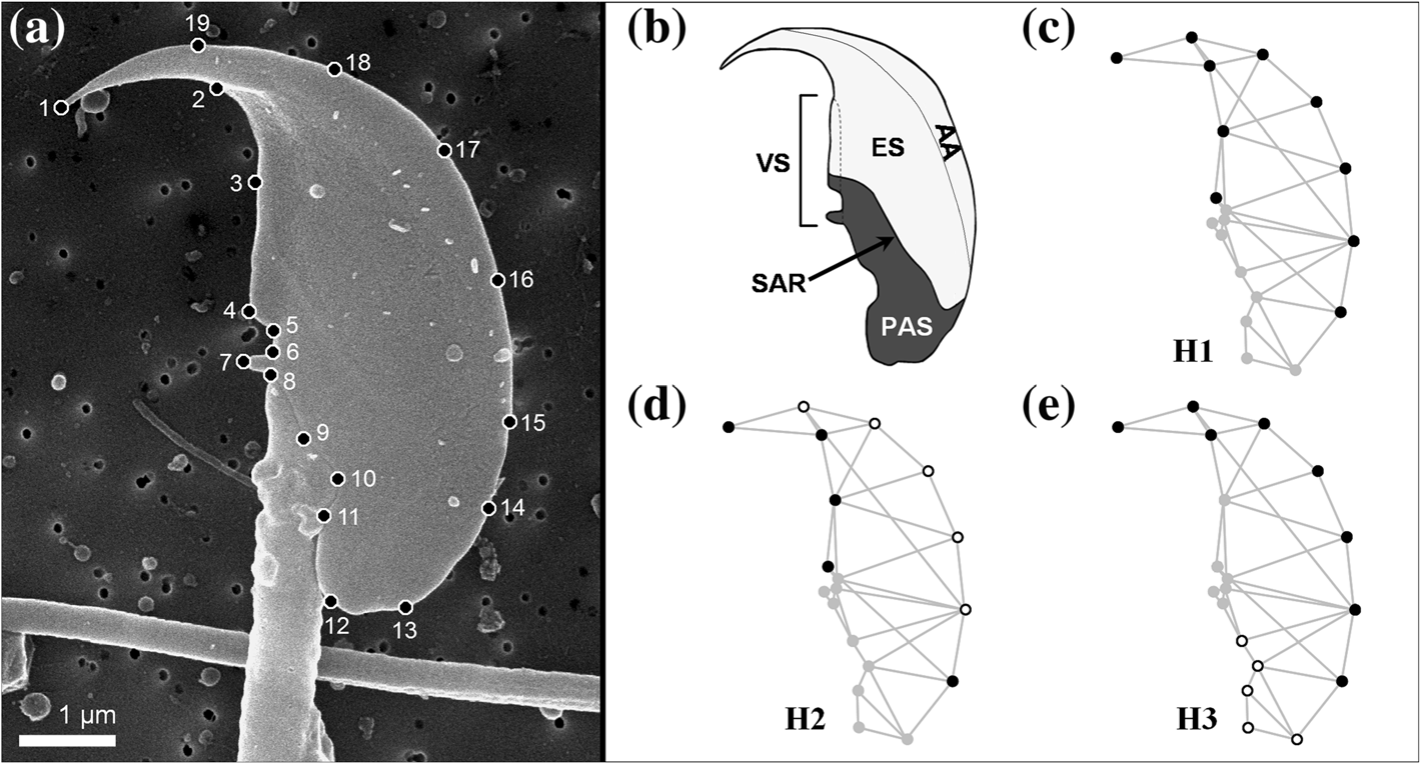
**House mouse sperm head. (a)** SEM image of the sperm head indicating the position of landmarks and semilandmarks. **(b)** Localization of the main regions of the sperm head: AA, anterior acrosome; ES, equatorial segment; SAR, sub-acrosomic ring; PAS, post-acrosomal sheath; VS, ventral spur. The acrosomal (APM) and post-acrosomal (PAPM) plasma membranes are shown in light and dark shading respectively. **(c**-**e)** Subsets of landmarks for hypotheses H1-H3.

The sperm of the western house mouse, *Mus musculus domesticus*, has a complex, flattened, hook-shaped head containing the cell nucleus [7]. Overlying the nucleus is the acrosome, which has two functional components: the anterior acrosome and the equatorial segment (Figure 1) [7,8]. Division at the organelle level also affects the organization of the plasma membrane and the cytoskeleton. The plasmalemma of the sperm head is structurally and functionally differentiated into two major domains, the acrosomal and the post-acrosomal plasma membranes, separated by the sub-acrosomal ring [8,9]. Beneath the plasma membrane there is a dense cytoskeletal mesh that forms the perinuclear theca (PT), which is also differentiated into two main domains: the outer periacrosomal layer and the post-acrosomal sheath [10,11]. In the latter domain, an area mainly composed by perforatorial proteins is distinguished: the ventral spur region [10,11]. In view of this noticeable compartmentation in the sperm head, the main goal of this study is to assess, for the first time, whether the structural and functional regionalization of the cellular components generates the variational modularity of cell shape. We used geometric morphometrics to test three hypotheses that divide the sperm head into different modules (Figure 1): i) acrosome and post-acrosome (H1); ii) anterior acrosome, equatorial segment and post-acrosome (H2); and iii) acrosome, post-acrosome and ventral spur (H3).

Previous studies in mice from the Robertsonian system found in Barcelona (BRbS) revealed that chromosomal rearrangements affect the size and the shape of the sperm head [12]. This Rb system represents a unique contact zone between standard (St) and Rb mice since there is no evidence of a Rb race in which a group of individuals from the same geographical area share a set of metacentrics in homozygous condition [13]. Diploid numbers range from 27 to 40 chromosomes, and seven different metacentrics (Rb3.8, 4.14, 5.15, 6.10, 7.17, 9.11 and 12.13) have been described up to now [14-16]. The relative stability of its metacentric staggered structure [16] as well as the phenotypic differences associated with karyotype detected in animals from this area [17] suggest the presence of partial barriers to gene flow. In this scenario, the study of the factors involved in the establishment of reproductive barriers between individuals may take on special relevance. Thus, assuming that Rb fusions could induce variations in the genotype-phenotype map of the sperm head [12], and that changes in the variational modularity patterns may play an important role in the evolvability of the sperm features, a second aim of this study is to evaluate the effect, if any, of the Rb translocations on the pattern of variational modularity of the western house mouse sperm head.

## Results and discussion

The Procrustes ANOVA performed on the replicated subsample showed highly significant differences between sperm heads (MS sperms = 0.000388, MS error = 0.000001, *P* < 0.0001). The mean squares for sperm head variation exceeded the mean squares for replicates by 388-fold, indicating low measurement error and consequently strong repeatability of the landmark location in the sperm head.

Fine morphological analysis of mouse sperm heads combining scanning electron microscopy and geometric morphometrics revealed significant allometry of cell shape (*P* < 0.001) with 11.8% and 8.6% of shape variation explained by changes in cell size in the St and Rb groups, respectively. Allometric shape changes affected all the landmarks to a similar degree and mainly involved a narrowing of the sperm head and a stretching of the hook. The existence of significant size-dependent shape changes is interesting because evidence of shape allometry at the cell level is very scarce. The precise mechanisms that underlie this association are unknown. However, recent studies have indicated a correlation between cell shape and growth [18,19], and between cell size and the behaviour of the cytoskeletal machinery [19]. Given that the cytoskeleton is mainly responsible for shaping the cell during growth, it is reasonable to suppose that these behavioural changes in the cytoskeletal machinery may be partly responsible for the association between size and shape of the cell. Because allometry represents a global integration factor, the residuals of the multivariate regression of the Procrustes coordinates onto log CS were used for further analyses.

**Figure 2:**
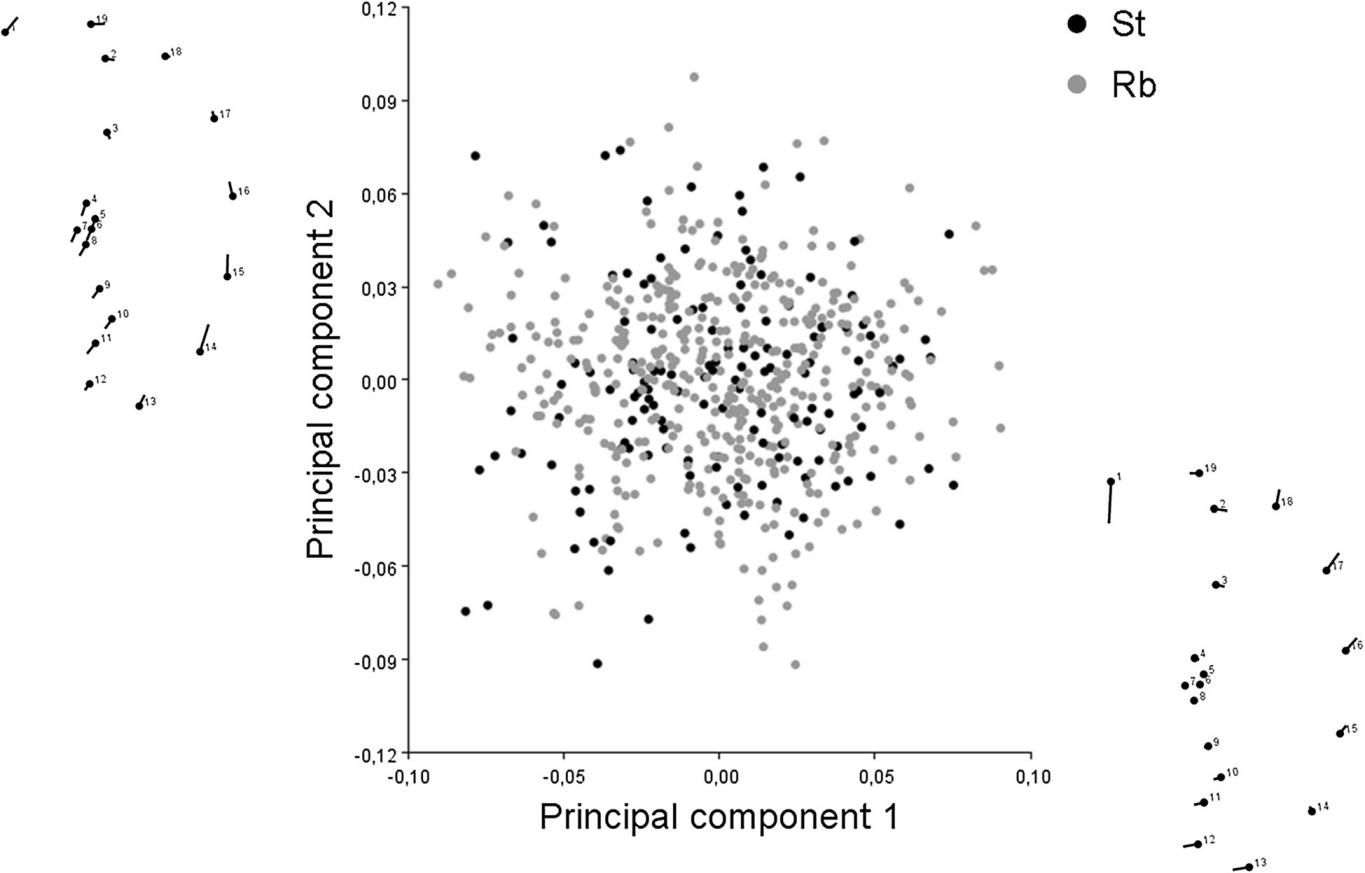
Principal component analysis and diagram of the shape changes associated with the first and second PCs.

**Figure 3:**
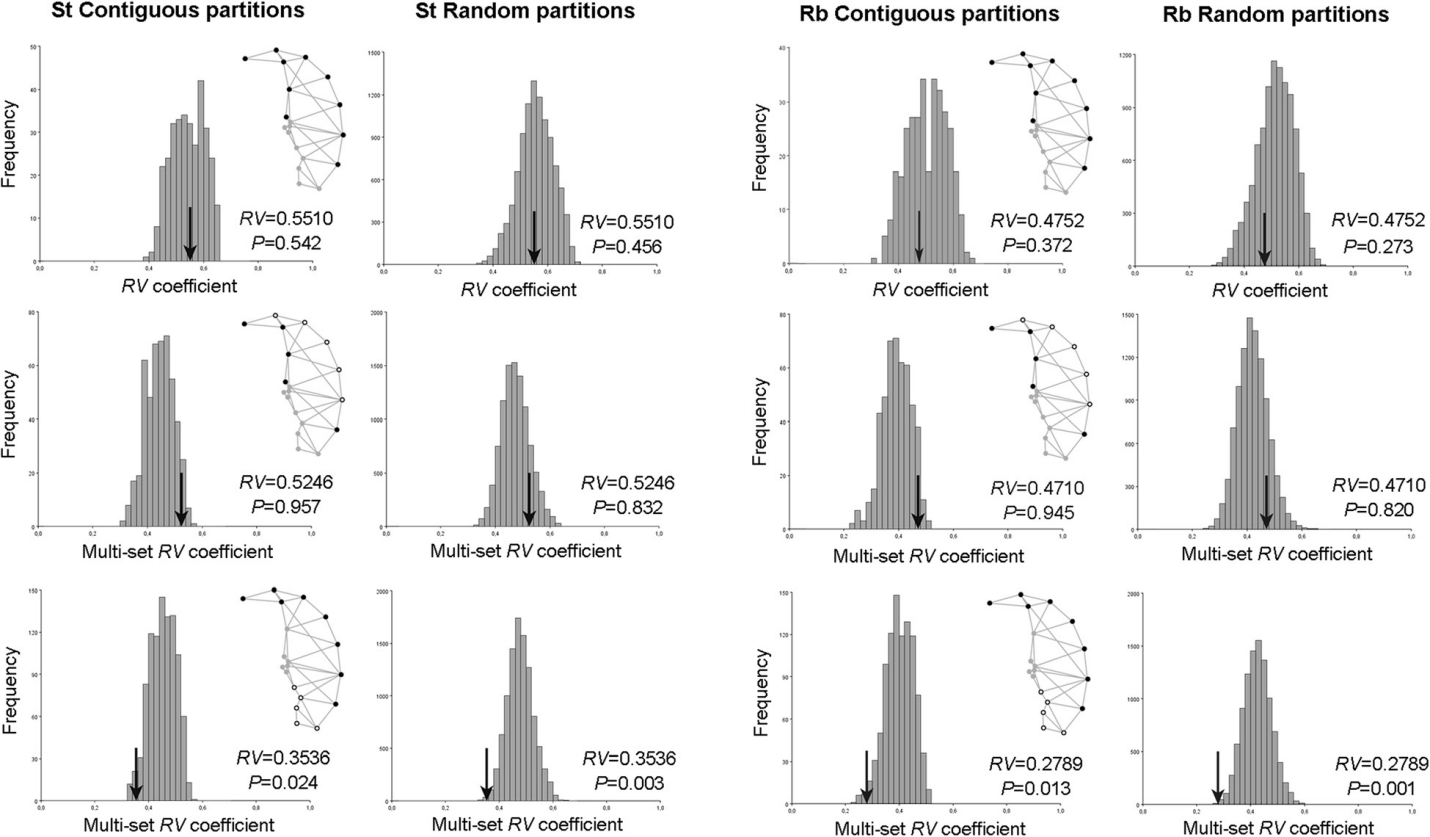
**Permutational distributions of the *RV *coefficients of contiguous and random partitions of the three modularity hypotheses (H1, upper panels; H2, middle panels; H3, lower panels) see Figure** 1**tested for standard (St) and Robertsonian (Rb) samples.** The arrow indicates the position of the *RV* coefficient of the hypothesized partition. *RV* coefficients and associated P-values (P) are indicated beside the graphs.

The PCA indicated that the first five PCs explained around 75% of the shape variation in both St and Rb mice (Figure 2; Table 1). The shape changes associated with PC1 were mainly concentrated in the hook and the convex side of the sperm head, while the changes associated with PC2 affected the ventral spur and post-acrosome (see Figure 2). The *RV* and multiset *RV* coefficients indicated that there was a low to moderate degree of covariation between the different regions of the sperm head (Figure 3). However, the only significant hypothesis when comparing the multiset *RV* value with permutational distributions was H3, which divides the sperm head in accordance with the structural division of the PT (Figure 3). The PT is a cytoskeletal structure with a central role in the morphogenesis and maintenance of sperm head shape [20,21]. While its heterogeneous composition is associated with the functional organization of the cell and the subdivision of the plasmalemma [11], our results indicate that the modularity of the sperm head shape is directly influenced by structural changes in the cytoskeleton. The functional division of the sperm head into three main regions (H2) seems not to play a direct role in the variational modularity of overall shape. Moreover, according to our results, the acrosome behaves as an integrated unit, while in the post-acrosomal region the ventral spur shows a high degree of autonomy. This modularity pattern was detected in data corrected and not corrected (results not shown) for allometry. This result indicates that size-dependent shape changes do not play a major role in the patterns of integration of the sperm head. The *RV* values obtained in the 2B-PLS analyses indicated a low strength of association between subsets of landmarks in H3 for all comparisons (Table 2). However, the percentages of covariation explained by the first PLS axis in the comparisons of the ventral spurs with the acrosomal and post-acrosomal domains were high, especially in the St group (Table 2). This result is congruent since the ventral spurs are considered to be specialized features integrated in the post-acrosomal sheath [10,20]. The high percentages of covariance explained by the first PLS axis in the St sperm head indicated that most of the covariation is concentrated in precise features of shape that change in a coordinated manner between modules. This is especially relevant in the comparison between the ventral spur and the acrosomal and post-acrosomal modules, where the first PLS explains around 85% of the covariation. These results suggest that the amount of covariation between certain pairs of modules may depend on the subdivision of the sperm plasma membrane, since the acrosomal domain overlies the whole acrosome and the upper ventral spur and the post-acrosomal domain involves the post-acrosome and the lower region of the ventral spur module [8,9]. In fact, the hypothesis testing the division of the sperm head into acrosomal and post-acrosomal modules (H1), although not significant, yielded an *RV* value lower than most of the alternative partitions, indicating a certain influence of the membrane domains. Conversely, the 2B-PLS revealed a different covariation pattern in Rb sperm heads. Several studies have evidenced that chromosomal rearrangements may induce changes in morphological covariation patterns through the rupture of genetic linkage groups and/or the occurrence of epistatic interactions between genes involved in the development of certain modules [22,23]. Under these circumstances, the variation in the genotype-phenotype map could explain the lower percentages of covariation among pairs of modules detected in Rb mice. However, the extent to which these differences affect the potential for evolutionary change remains to be elucidated.

**Table 1:** Eigenvalues and percentages of variance and cumulative variance explained by the first ten principal components (out of 34) of the PCA obtained with the residuals from the multivariate regression analysis

	**St**			**Rb**		
**PC**	**Eigenvalues**	**Variance (%)**	**Cumulative (%)**	**Eigenvalues**	**Variance (%)**	**Cumulative (%)**
1.	0.00144	25.155	25.155	0.00147	27.376	27.376
2.	0.00107	18.594	43.749	0.00090	16.700	44.076
3.	0.00076	13.238	56.987	0.00067	12.518	56.594
4.	0.00056	9.731	66.718	0.00055	10.288	66.883
5.	0.00046	8.008	74.726	0.00042	7.823	74.706
6.	0.00027	4.708	79.434	0.00025	4.701	79.407
7.	0.00022	3.907	83.341	0.00023	4.306	83.713
8.	0.00019	3.371	86.711	0.00018	3.419	87.132
9.	0.00015	2.629	89.341	0.00013	2.431	89.563
10.	0.00014	2.383	91.723	0.00012	2.205	91.768

*Abbreviations*: *PC*, Principal components; *St*, standard sperm heads; *Rb*, Robertsonian sperm heads.

**Table 2:** Results of 2B-PLS analyses for standard (St) and Robertsonian (Rb) samples

**Group**	**Blocks**	***RV***	**P**	**%Total Cov PLS1**	**Corr PLS1**	**P**
	AC vs PA	0.034	0.0071	61.8	0.282	0.0001
St	AC vs VS	0.035	0.0064	86.3	0.330	0.0001
	PA vs VS	0.067	0.0001	83.7	0.321	0.0001
	AC vs PA	0.045	0.0001	55.5	0.307	0.0022
Rb	AC vs VS	0.027	0.0043	56.9	0.336	0.0001
	PA vs VS	0.031	0.0001	52.2	0.289	0.0001

For each two-block comparison we display the *RV* coefficient; the percentage of total covariation explained by the first PLS axis; the correlation between the 2B-PLS1 scores; and the associated P-values (P).

## Conclusions

Our results reveal for first time the existence of variational modularity in a cellular structure such as the house mouse sperm head and highlight the important role of the cytoskeleton in maintaining the shape of the cell. The presence of Rb translocations did not affect the variational modularity pattern. However, the lower percentages of shape covariation between pairs of modules in Rb sperms heads suggest a certain influence of the Rb rearrangements. Understanding the mechanisms that alter covariation between phenotypic traits in the sperm head is an aspect of great importance given its possible effect on the evolvability of these specialized cells. However, the extent to which these changes affect sperm fertilization success is a subject for further studies.

## Methods

Thirty-one live-trapped males in the BRbS were used for analyses. Karyotypes were obtained from a suspension of bone marrow cells, following Ford [24]. Metaphase chromosome spreads were stained by a G-banding method [25]. Chromosome identification was performed following the Committee on Standardized Genetic Nomenclature for mice [26]. The left caudate epididyme from 13 St and 18 Rb house mice was dissected and disaggregated in 5 ml of phosphate buffer (PB) 0.1 M at room temperature. After homogenization, 1 ml of sperm solution was filtered through a nucleopore membrane (0.2 μm) and fixed in 2.5% glutaraldehyde, 2% paraformaldehyde and PB 0.1 M solution. Samples were then rinsed in PB 0.1 M, postfixed in 1% osmium tetraoxide, rinsed in PB 0.1 M, dehydrated in graded series of ethanol and dried by the critical-point method. Membranes were observed in an S-570 scanning electron microscope (SEM; Hitachi Ltd.) at an accelerating voltage of 15 kV. From each individual, an average of 20 sperm heads in a horizontal plane, with the hook orientated to the left side and without evident structural alterations were randomly captured (Figure 1).

To determine the form of the sperm heads, sixteen landmarks and three semilandmarks were digitized using the tpsDig2 software [27] (Figure 1). The criteria used for the landmark assignation were the following: (1) top of the hook, (2) point where the hook and the upper ventral spur overlap, (3) prominence in the axis of the upper ventral spur, (4 and 7) top of the upper and lower ventral spurs, (5 and 6) inner distance between the ventral spurs, (8–11) insertion edge of the sperm head with flagellum, (12 and 13) terminal edges of the post-acrosomal sheath, (14,15 and 19) basal and apical ridge of the equatorial crest. The semilandmarks (points 16–18) were digitized as equidistant points by the tpsDig2 ‘resample curve by length’ option. Measurement error is an important source of variation affecting morphometric data that can increase the likelihood of type II errors and lead to biased results [28,29]. In order to evaluate the impact of measurement error in the current set of landmarks around the sperm head, in a subsample of 40 images all landmarks were digitized three times. Geometric morphometrics and modularity analyses were performed using the routines implemented by MorphoJ software [30]. Shape variation in the landmark configurations was obtained by the full Procrustes fit and the orthogonal projection to the tangent space [31]. Size was defined as centroid size (CS) [32]. In the replicated subsample, a Procrustes ANOVA comparing variation among and within sperm heads was performed to obtain the measurement error associated with landmark location [33,34]. Given that variation between sperm heads clearly exceeded that of measurement error (see Results) subsequent analyses were based on a single digitization of landmarks per head. Shape allometry, the scaling of shape with size, may conceal the patterns of modularity [35]; thus, the dependence of shape on size was calculated by means of a linear multivariate regression of the Procrustes coordinates onto the logarithm of CS [35]. Statistical significance was obtained using a permutation test with 10,000 iterations under the null hypothesis of size and shape independence [36]. The residuals obtained in the multivariate regression analyses were used for subsequent analyses [35]. First, principal component analysis (PCA) was performed with the covariance matrix of the residuals. Then, the division of the sperm head into three different sets of morphological modules was tested (Figure 1). To measure the covariation between the hypothesized sets of landmarks, the *RV* coefficient or the multi-set *RV* coefficient was obtained [35]. To test for modularity, this value was compared with the distribution of *RV* values of all the alternative partitions of spatially contiguous subsets of landmarks (adjacency graphs in Figure 1) containing the same number of landmarks as the hypothesized partitions and with 10,000 random partitions [35]. Finally, we used a two-block partial least square (2B-PLS) to examine covariation between the detected modules [37,38]. Because of differences in mice karyotypes, these analyses were performed separating the sample into two different chromosomal groups: i) St, sperms produced by animals with 40 chromosomes and ii) Rb, sperms from animals ranging from 30 to 39 chromosomes.

Permission to capture was granted by the Departament de Medi Ambient of the Generaltitat de Catalunya (Spain). Animals were handled in compliance with the guidelines and ethical approval by the Comissió d’Ètica en l’Experimentación Animal y Humana (CEEAH) of the Universitat Autònoma de Barcelona and by the Departament d’Agricultura, Ramaderia, Pesca, Alimentació i Medi Natural (Direcció General de Medi Natural i Biodiversitat) of the Generalitat de Catalunya (reference of the experimental procedure authorization: DAAM 6328).

## Competing interests

The authors declare that they have no competing interests.

## Authors’ contributions

NM carried out the fieldwork, the karyotype of the animals and data acquisition. She also has participated to the design of the study, analysis and interpretation of data, and drafting the manuscript. FM conceived the study, carried out the statistical analysis, interpretation of data, and drafted the manuscript. MJL and JV participated in the design of the study and revised the manuscript. JV coordinated the study and the research project in which it is included. All authors read and approved the final manuscript.
